# Predictors of Response to Induction Therapy with Ustekinumab in Patients with Ulcerative Colitis: Results from a National Study in Greece

**DOI:** 10.3390/diseases14040149

**Published:** 2026-04-19

**Authors:** Konstantina Chalakatevaki, Georgios Kokkotis, Maria Gazouli, Stratigoula Sakellariou, Ioannis Vamvakaris, Alexandros Chatzidakis, Gerasimos Gerasimatos, Maria Kalogirou, Kanellos Koustenis, Dimitra Lazou, Afroditi Orfanidou, Maria Palatianou, Evgenia Papathanasiou, Andreas Psistakis, Christos Sotiropoulos, Evaggelia Anagnostopoulou, Konstantinos Argyriou, Matina-Lydia Chatzinikolaou, Kalliopi Foteinogiannopoulou, Olga Giouleme, Andreas Kapsoritakis, Pantelis Karatzas, Konstantinos Karmiris, Nikolaos Kiriakos, Ioannis Koutroubakis, Christos Liatsos, Aikaterini Mantaka, Gerasimos Mantzaris, Panagiotis Markopoulos, Georgios Michalopoulos, Spiros Michopoulos, Dimitrios Polymeros, Konstantinos Soufleris, Georgios Theocharis, Angeliki Theodoropoulou, Eftychia Tsironi, Maria Tzouvala, Nikos Viazis, Eirini Zacharopoulou, Evanthia Zampeli, Giorgos Bamias

**Affiliations:** 1 3rd Academic Department of Internal Medicine, University of Athens-Sotiria Hospital-GI Unit, 11527 Athens, Greece; kchalakatevaki@gmail.com (K.C.); gkokkot@gmail.com (G.K.); 2 Laboratory of Biology, Department of Basic Medical Sciences, National and Kapodistrian University of Athens, 11527 Athens, Greece; maria.gazouli@gmail.com; 3 Pathology Laboratory, National and Kapodistrian University of Athens, 11527 Athens, Greece; sakellarioustrat@yahoo.gr; 4 Pathology Laboratory, Sotiria Hospital, 11527 Athens, Greece; i.vamvakaris@yahoo.gr; 5 Department of Gastroenterology, Attikon University Hospital, 12462 Athens, Greece; alexandroshatzidakis@gmail.com (A.C.); dimpolymeros@yahoo.com (D.P.); 6 Department of Gastroenterology, General Hospital G. Gennimatas, 11527 Athens, Greece; gerasimatos.ger@gmail.com (G.G.); gmicha78@hotmail.com (G.M.); 7 2nd Propaedeutic Department of Internal Medicine, Aristotle University of Thessaloniki, 54642 Thessaloniki, Greece; maria.kalogi32@gmail.com (M.K.); olga.giouleme@gmail.com (O.G.); 8 Department of Gastroenterology, Evangelismos Hospital, 10676 Athens, Greece; k.koustenis@yahoo.gr (K.K.); gjmantzaris@gmail.com (G.M.); nikos.viazis@gmail.com (N.V.); 9 Department of Gastroenterology, Theagenio Cancer Hospital of Thessaloniki, 54639 Thessaloniki, Greece; dimlazou@hotmail.com (D.L.); ksoufleris@yahoo.gr (K.S.); 10 Department of Gastroenterology, General Hospital Laiko, 11527 Athens, Greece; afroditi.orfanidou@gmail.com (A.O.); panteliskaratzas@gmail.com (P.K.); 11 Department of Gastroenterology, General Hospital of Nikea and Pireaus, 18454 Athens, Greece; marela_p@yahoo.gr (M.P.); tzouvalam@gmail.com (M.T.); eirinizachar@gmail.com (E.Z.); 12 Department of Gastroenterology, General Hospital Alexandra, 11528 Athens, Greece; evgeniapapathanasiou@gmail.com (E.P.); michosp5@gmail.com (S.M.); evazamb@gmail.com (E.Z.); 13 Department of Gastroenterology, Venizeleio General Hospital, 71409 Heraklion, Greece; andrew_psistos@hotmail.com (A.P.); kkarmiris@gmail.com (K.K.); angelikitheodoropoulou@yahoo.co.uk (A.T.); 14 Department of Gastroenterology, General University Hospital of Patras, 26504 Patras, Greece; cr.sotiropoulos@hotmail.com (C.S.); giorgistheocharis@gmail.com (G.T.); 15 Department of Gastroenterology, Saint George General Hospital of Chania, 73300 Chania, Greece; evanagnost@hotmail.com (E.A.); katmant@gmail.com (A.M.); 16 Department of Gastroenterology, University General Hospital of Larissa, 41334 Larissa, Greece; kosnar2@yahoo.gr (K.A.); kapsoritakis@uth.gr (A.K.); 17 Metropolitan General Hospital, 15564 Athens, Greece; matinahatzinicolaou@gmail.com; 18 Department of Gastroenterology, University General Hospital of Heraklion, 71500 Heraklion, Greece; drfpopi@hotmail.com (K.F.); ikoutroubakis@gmail.com (I.K.); 19 Department of Gastroenterology, 401 General Military Hospital, 11525 Athens, Greece; nkyriakos79@gmail.com (N.K.); cliatsos@yahoo.com (C.L.); 20 Department of Gastroenterology, Metaxa Cancer Hospital of Athens, 18537 Athens, Greece; panosmarkmd@gmail.com (P.M.); etsironi@yahoo.com (E.T.)

**Keywords:** biomarkers, efficacy, mucosal expression

## Abstract

Background/Objectives: Ustekinumab has been approved for the treatment of moderate to severe ulcerative colitis. Real-world data regarding its efficacy and the discovery of predictive factors of response need to be studied further. We aimed to evaluate the efficacy and identify predictors of response to induction treatment with ustekinumab in patients with ulcerative colitis. Methods: This is a multicenter, prospective cohort study. Clinical response (CR) at week 16 was the primary endpoint, and steroid-free clinical remission (SFCRem) and endoscopic response were the secondary endpoints. Baseline histology, mucosal gene expression, and pharmacokinetics were studied for their effect on response to treatment. Results: We included 123 patients (mean age = 50.3 years). CR was recorded in 70.8% (75/106), SFCRem in 48% (59/123), endoscopic improvement in 71.4% (40/56), and mucosal healing in 28.6% (16/56). Higher PRO-stool frequency (OR = 0.49, *p* = 0.027), concomitant use of 5-ASA (OR = 3.69, *p* = 0.021), platelet number of ≥284 × 10^9^/L (OR = 6.52, *p* = 0.001) at baseline, and a drop in the total count of platelets by 10^8^/L (OR = 1.23, *p* = 0.022) at week 8 were independently associated with CR. Elevated trough levels of ustekinumab at week 16 were associated with a higher probability of endoscopic improvement (median difference = 3784 ng/mL, *p* = 0.013), with an optimal cut-off value of 3500 ng/mL (AUC = 0.82, 95% CI: 0.66–0.96). Increased mucosal mRNA expression for *IL-23* (*p* = 0.007) and *IL-23R* (*p* = 0.031) at baseline was associated with increased probability of CR. Higher continuous Geboes scores at baseline were associated with a lower probability of CR (OR = 0.80, *p* = 0.045), with an optimal cut-off value of 14 (AUC = 0.75, 95% CI: 0.57–0.93). Conclusions: Clinical, laboratory, and molecular markers may identify patients with ulcerative colitis who are more likely to respond to ustekinumab.

## 1. Introduction

Ulcerative colitis (UC) is one of the two major forms of inflammatory bowel disease (IBD). It is characterized by chronic, remitting, and relapsing intestinal inflammation that typically localizes at the colonic mucosa, spreading proximally from the anal verge in a continuous manner [1]. Persistence of inflammation is associated with major adverse outcomes in patients with UC, such as the development of colon cancer and/or colectomy [2,3]. Furthermore, it may severely affect the quality of life of patients due to the persistence of symptoms such as diarrhea, fecal urgency, and incontinence. Finally, it leads to frequent utilization of health system resources and, in association with the worldwide increase in the prevalence of IBD, may pose a significant financial burden both at the individual and public levels. These facts emphasize the need to develop therapies with the ability to effectively control inflammation and prevent long-term complications and disability in patients with UC. Accordingly, the current treatment paradigm for UC supports the timely introduction of advanced therapies, soon after failure of therapy with 5-ASA to longitudinally control intestinal inflammation [4]. Such therapies include monoclonal antibodies that target cytokines like TNF-α, the p19 chain of IL-23, or the common p40 chain of IL-23 and IL-12 and small molecules that inhibit signaling via JAK kinases or modulate the function of S1PR [5].

Ustekinumab (UST) is a monoclonal antibody that binds to the p40 chain, thus effectively inhibiting signaling via both IL-12/IL-12R and IL-23/IL-23R. UST is currently approved for the treatment of moderate-to-severe UC, in addition to other indications, which include Crohn’s disease (CD), psoriasis, and psoriatic arthritis. Approval for UC was granted based on the results of the regulatory trial UNIFI that demonstrated superiority over placebos in various efficacy outcomes with no safety risks [6]. Nevertheless, it is well-accepted that, given the strict inclusion and exclusion criteria, regulatory trials are not representative of real-world patient populations [7]. This highlights the need for obtaining and reporting data from patients who are treated with advanced therapies, outside the confines of regulatory trials. So far, there are few such reports regarding the use of UST in real-world populations [8]. Furthermore, the rapidly increasing number of diverse therapeutic options for patients with UC has also brought about the need to recognize patient profiles that will benefit the most from each treatment and specify their safety and efficacy.

In the present study, we report real-world findings from a large, multicenter, national cohort of patients with UC who commenced treatment with UST. The overall aim of our study is to prospectively follow up patients over 2 years and define long-term efficacy with UST. Data collection was concluded in January 2026. Herein, we present our findings of the 16-week induction period. Based on our analysis, we confirm high response rates to UST for several clinical and endoscopic outcomes at week 16. More importantly, we report that response to induction treatment with UST may be predicted at baseline based on the presence of clinical and histological factors. We demonstrate exposure/effect associations for UST as indicated by higher drug levels in patients with a favorable endoscopic outcome. Finally, we show that UST efficacy may depend on the abundance of the treatment target at baseline, as it is signified by elevated mucosal *IL-23* and *IL-23R* mRNA.

## 2. Materials and Methods

### 2.1. Study Design

This is an ongoing prospective cohort study with enrollment commencing in March 2020 and concluding in January 2024. This study was conducted in accordance with the Declaration of Helsinki and approved by the Ethics Committee of the National and Kapodistrian University of Athens, and informed consent was obtained from all subjects involved in this study. The entry point is considered the day of the initial weight-adjusted intravenous dose infusion of UST, and week 16 is defined as the end of the induction regimen, where the first subcutaneous injection of 90 mg is administered. Each patient was followed up for 2 years to record the efficacy and adverse outcomes of UST, finalizing data collection in January 2026. Clinical, endoscopic, and biochemical activity was evaluated upon enrollment and sequentially in specified follow-up time points, namely week 8 [W8] and week 16 [W16], and evaluations were carried out for week 54 [W54], week 80, and week 108. Herein, only baseline, W8, and W16 data will be discussed, as shown in Supplementary Figure S1.

### 2.2. Study Population

This study includes Greek patients with UC who received treatment with UST between March 2020 and January 2024 in 16 centers named in Supplementary Table S1. Inclusion criteria were defined as patients ≥ 17 years old with moderate-to-severe UC who commenced UST during the set timeline. Exclusion criteria were defined as patients younger than 17 years old, Crohn’s disease or unclassified IBD, and treatment commencement outside the set timeline.

### 2.3. Study Endpoints

Herein, we report results at the end of induction [W16]. The primary endpoint of the present analysis is clinical response [CR] at W16, defined as a drop of at least 50% in PROs [“stool frequency” + “rectal bleeding”] (extensively described in the “data collection” paragraph) compared to baseline values. Secondary endpoints are corticosteroid-free clinical remission [SFCRem] (defined as partial MAYO score < 2, “stool frequency” = 0, and “rectal bleeding” = 0, without concomitant use of corticosteroids), endoscopic improvement [EI], mucosal healing [MH] defined as a complete absence of mucosal inflammation at endoscopy, histologic remission [HR] defined as continuous Geboes scores of ≤6, and definition of predictors of response to UST at W16.

### 2.4. Data Collection

Age, gender, and disease-related data were collected before UST initiation. Namely, we recorded the disease duration, disease extension according to the Montreal classification (E1: ulcerative proctitis; E2: left-sided [distal] colitis; E3: extensive colitis [pancolitis]) [9], the presence of extra-intestinal manifestations (EIMs), and prior and concomitant treatments of UC.

For the evaluation of clinical activity, we used the partial MAYO (pMAYO) score, which consists of 3 parameters ranging from 0 to 9 [a higher score represents higher disease activity]. These 3 parameters are the patient-reported outcome (PRO) of “Stool frequency”, the PRO of “Rectal bleeding”, and the physician’s global assessment [PGA], ranging from 0 to 3. In detail, pMAYO is scored: “Stool frequency”: 0 = normal, 1 = 1–2 stool/day more than normal, 2 = 3–4 stool/day more than normal, and 3 = 5+ stool/day more than normal; “Rectal bleeding”: 0 = none, 1= visible blood with stool less than half the time, 2 = visible blood with stool half of the time or more, and 3 = passing blood alone; “Physician’s Global Assessment”: 0 = normal, 1 = mild disease, 2 = moderate disease, and 3 = severe disease [10].

Biochemical activity was evaluated with hemoglobin [mg/dL], platelets [per microliter], white blood cells [per microliter], C-reactive protein [mg/L], and albumin [mg/dL].

For the assessment of endoscopic activity, the MAYO endoscopic score and the Ulcerative Colitis Endoscopic Index of Severity (UCEIS) [11] were recorded. MAYO endoscopic scores were used to evaluate the colonic mucosa and can be scored as 0 = normal, 1 = mild disease [erythema, decreased vascular pattern, mild friability], 2 = moderate disease [marked erythema, absent vascular pattern, friability, erosions], and 3 = severe disease [spontaneous bleeding, ulceration]. UCEIS is the sum of three separate parameters of the mucosa: vascular pattern [0 = normal, 1 = patchy obliteration, 2 = obliteration], bleeding [0 = none, 1 = mucosal, 2 = luminal mild, 3 = luminal moderate/severe], and erosions and ulcers [0 = none, 1 = erosions, 2 = superficial ulcer, 3 = deep ulcer]. Both scores correlate with higher endoscopic activity.

All biopsies were examined by the same experienced pathologist, and histologic activity was evaluated with the use of the continuous Geboes histology score. The Geboes score grades the following specific features: architectural changes (Grade 0), chronic inflammatory infiltrate (Grade 1), eosinophils in lamina propria (Grade 2A), neutrophils in lamina propria (Grade 2B), neutrophils in epithelium (Grade 3), crypt destruction (Grade 4), and erosions and ulcerations (Grade 5). Each feature has four to five grades. The continuous Geboes score is calculated by adding the sub-scores, and it can range from 0 to 22, with higher values found in more severe disease; a value of ≤6 is considered as HR [12].

Additional biopsies were obtained before UST initiation and stored in RNA later (Macherey-Nagel, Düren, Germany) at −80 °C to perform real-time reverse transcription polymerase chain reaction (RT-PCR) and compare *IL12RB1*, *IL12RB2*, *IL12*, *IL23R*, and *IL23* mRNA expressions in patients who achieved and did not achieve the primary and secondary endpoints. Total RNA was extracted from preserved mucosal biopsies using the Qiagen AllPrep RNA/DNA Mini Kit (Qiagen, Hilden, Germany). cDNA was prepared using the RT^2^ First Strand Kit (Qiagen, Hilden, Germany) according to the manufacturer’s instructions. Assessment of the *IL12RB1*, *IL12RB2*, *IL12*, *IL23R*, and *IL23* mRNA levels was performed by employing glyceraldehyde-3-phosphate dehydrogenase (GAPDH) expression levels as a reference gene. The primer sequences used are demonstrated in Supplementary Table S2. Real-time polymerase chain reaction (PCR) was performed in an SaCycler-96 system (Sacace Biotechnologies, Como, Italy) as follows: initial denaturation for 2 min at 50 °C and for 10 min at 95 °C, followed by 40 cycles of PCR (95 °C for 15 s; 60 °C for 1 min). Reactions were performed in duplicate, with specific primer sets and PCR master mix (KAPA SYBR FAST qPCR Kit) according to the manufacturer’s instructions. Data were analyzed using the comparative CT method for the relative quantitation of results.

UST trough levels (ng/mL) were measured in patients’ serum at W16 using an ELISA Kit (HUMB00020) (Assay Genie, Dublin, Ireland) according to the manufacturer’s instructions to identify probable correlations with the primary and secondary endpoints.

### 2.5. Statistical Analysis

Statistical analysis was performed with the statistical package SPSS 23 (IBM, Armonk, NY, USA). For categorical variables, the total count and percentages are presented. For continuous variables that are normally distributed, “Mean value” and “Standard Deviation” are presented, while for those not normally distributed, the “Median” and “Range” are presented. Normal distributions were assessed using the Shapiro–Wilk test and were further confirmed using histograms. For the comparison of continuous variables, the parametric paired-sample *t*-test and non-parametric Wilcoxon test were performed. The non-parametric chi-square test was used for the comparison of categorical outcomes. Receiver operating characteristic (ROC) curves were created to identify the optimal cut-off values. Univariate logistic regression models were performed for the identification of potential predictors of the primary outcome of clinical response, with a cut-off *p*-value = 0.1. Factors of potential significance were later included in multiple logistic regressions to identify independent associations. A *p*-value = 0.05 was used as a threshold of statistical significance.

## 3. Results

### 3.1. Study Population

At the end of recruitment in January 2024, a total of 155 patients were enrolled in the study. All patients received an intravenous induction dose of approximately 6 mg/kg and the first 90 mg sc injection of UST, and all have reached W16 of follow-up. The 2-year follow-up of the study was completed by January 2026.

The baseline characteristics of our cohort are presented in Table 1. Most patients were male (63%) with a mean age of 50.3 years and a median disease duration of 8 years. A history of EIMs was reported by 39 patients [32%], while in 17 [14%], EIMs were active at UST initiation. Regarding prior treatments, 106 patients [86%] were exposed to corticosteroids, 118 [96%] to 5-ASA, 46 [37%] to azathioprine, and 5 [4%] to methotrexate. Eighty-seven [71%] had received advanced therapy with biologics, with 38 [31%] having been exposed to ≥2 biologics before UST initiation. At induction, corticosteroids were used as concomitant treatment in 62 [50%] of the cases, 5-ASA in 85 [69%], azathioprine in 12 [10%], and methotrexate in 1 [1%].

### 3.2. Induction Therapy with UST Is Associated with High Rates of Clinical and Endoscopic Outcomes at Week 16

**Figure 1:**
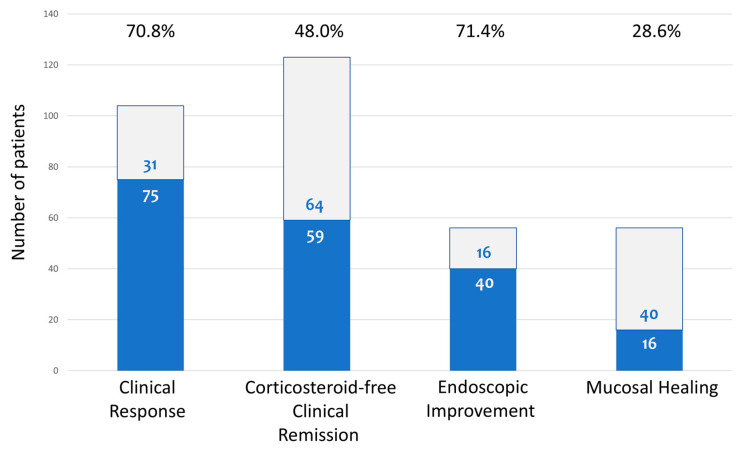
Clinical and endoscopic outcomes at week 16. Percentages of patients who achieved steroid-free clinical remission, clinical response, endoscopic improvement, and mucosal healing at the end of induction therapy (week 16).

At W16, adequate data were available for 123 participants. Therefore, statistical analysis was performed for those patients and presented herein. At W16, 59/123 patients [48.0%] were in SFCRem. Among patients with clinically active disease at baseline [n = 106], CR was reported by 75 [70.8%]. Fifty-six patients had paired endoscopic evaluations both at baseline and at wk16. Among those, 40 patients [71.4%] had EI, and 16 [28.6%] had MH (Figure 1).

### 3.3. Clinical and Laboratory Markers at Baseline and Early On-Treatment [8w] Predict CR to UST at W16

Next, we sought to identify patient- or disease-related factors at baseline that may be associated with CR with respect to induction therapy with UST, which was the primary endpoint of our study. As shown in Table 2, in the univariate logistic regression analysis, CR at W16 was negatively associated with prior exposure to corticosteroids, prior exposure to vedolizumab, higher baseline PRO-stool frequency, and concomitant use of corticosteroids, and it was positively associated with the concomitant use of 5-ASA and a platelet count of ≥284 × 10^9^/L. Subsequently, these factors were analyzed together in a multivariate model, in which higher PRO-stool frequency (OR = 0.49, 95% CI = 0.26–0.92, *p* = 0.027), concomitant use of 5-ASA (OR = 3.69, 95% CI = 1.20–11.17, *p* = 0.021) and platelet count of ≥284 × 10^9^/L (OR = 6.52, 95% CI = 2.08–20.46, *p* = 0.001) remained independently associated with CR at W16.

Furthermore, we also evaluated whether early W8 alterations of clinical scores and/or laboratory values compared to baseline values had a predictive value for accomplishing CR at W16. In the univariate logistic regression analyses, CR at W16 was positively associated with a decrease in PRO-rectal bleeding by 1 point, the concomitant use of 5-ASA, and a decrease in the total count of platelets by 10^8^/L at W8 compared to the baseline (Table 3). Subsequently, these factors were analyzed together in a multivariate model, in which only the drop in the total count of platelets by 10^8^/L (OR = 1.23, 95% CI = 1.03–1.47, *p* = 0.022) remained independently associated with CR at W16.

### 3.4. Higher Serum Concentration Levels of UST at W16 Are Associated with Better Endoscopic Outcomes

**Figure 2:**
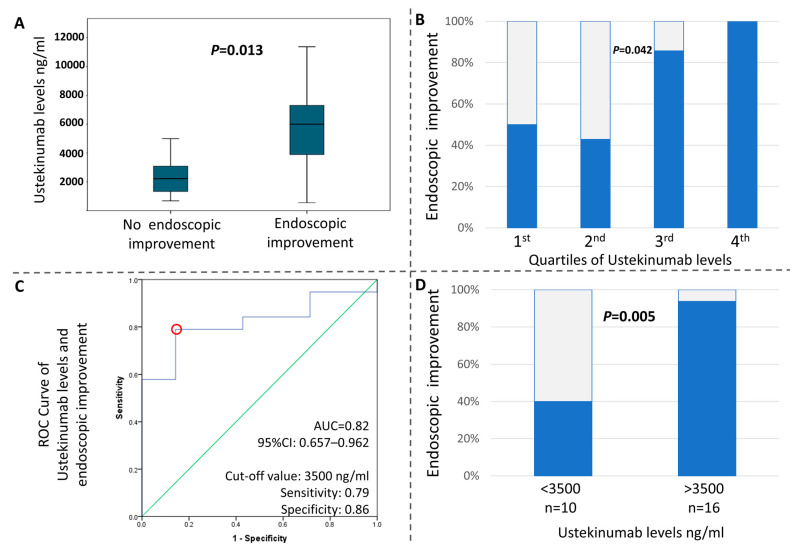
Exposure–response associations at week 16. (**A**) Endoscopic improvement was strongly associated with significantly higher ustekinumab trough levels. (**B**) Distribution of patients into 4 quartiles according to their ustekinumab trough levels. The higher quartile was associated with greater endoscopic improvement. (**C**) Receiver operating characteristic curve analysis. A cutoff value of 3500 ng/mL for ustekinumab trough levels was found to distinguish between endoscopic improvement or the lack of it. (**D**) Ustekinumab trough levels over 3500 ng/mL were associated with significantly higher rates of endoscopic improvement.

We next tested whether an exposure/efficacy effect for UST was present in our study. In support of this, our analysis showed that the serum trough levels of UST significantly correlated with the endoscopic activity of the disease at W16. In fact, the Mann–Whitney U test exhibited *p* = 0.013, as shown in Figure 2A. Additionally, we distributed the patients into quartiles according to their trough levels and calculated the respective rates of EI. We found that a higher quartile was associated with higher endoscopic response rates (1st: 50%; 2nd: 57.1%; 3rd: 85.7%; 4th: 100%, *p* = 0.042) (Figure 2B). We then performed an ROC curve analysis to identify a cut-off value for UST trough levels, which distinguishes the patients with EI (AUC = 0.82, 95% CI:0.66–0.96). Based on the curve and coordination table, we identified 3500 ng/mL as the value with the optimal combination of specificity (0.86) and sensitivity (0.79) for discriminating between EI or the absence of it (Figure 2C). Patients with UST trough levels over 3500 ng/mL achieved significantly higher rates of EI than those with less than 3500 ng/mL (chi-square *p* = 0.005) (Figure 2D).

### 3.5. Increased Mucosal Expression of IL-23 and IL-23R mRNA at Baseline Is Associated with Increased Probability of CR at W16

**Figure 3:**
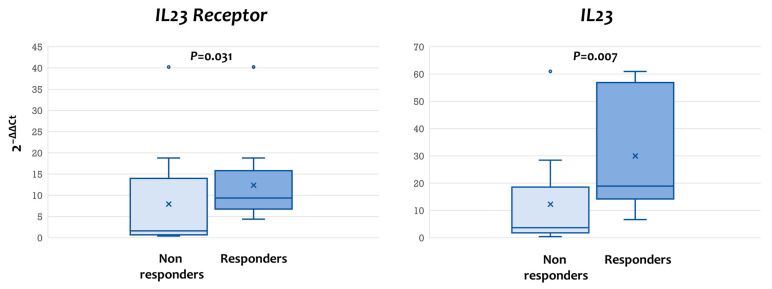
Elevated pre-treatment mucosal expression of IL-23 and IL-23R mRNA is predictive of response to therapy with ustekinumab in ulcerative colitis. The mRNA expression for IL-23 and IL-23R was quantified in total RNA extracted from endoscopically obtained biopsies before treatment with ustekinumab. Response to induction therapy with ustekinumab was significantly higher in patients with elevated expression of *IL-23* and *IL-23R* mRNA at baseline.

UST is a monoclonal antibody that binds the p40 chain, which is common for cytokines IL-12 and IL-23. We hypothesized that, by studying the baseline mucosal expression of *IL-12* and *IL-23* and their respective receptors in relation to responses to induction treatment with UST, we could conclude which target pathway may be more important for the efficacy of this biologic agent. Our study showed that the mRNA transcripts for both *IL-23R* and *IL-23* were highly elevated in patients who responded to UST than those who did not (*IL23R:* responders, median 2^−ΔΔCt^ =9.38, IQR = 6.75–15.81 vs. non-responders, median = 1.61, IQR = 0.70–13.98, *p* = 0.031; *IL23:* responders, median = 18.95, IQR = 14.22–56.89 vs. non-responders, median = 3.69, IQR = 1.77–18.54, *p* = 0.007) (Figure 3). On the other hand, no statistically significant difference was observed in the mRNA expression of *IL12RB1*, *IL12RB2*, and *IL12* (Supplementary Figure S2). Taken together, the results show that the IL-23/IL-23R signaling pathway may be the critical one for the anti-inflammatory effect of UST.

### 3.6. Higher Histologic Severity at Baseline Predicts Lower Clinical and Endoscopic Response Rates to Treatment with UST

**Figure 4:**
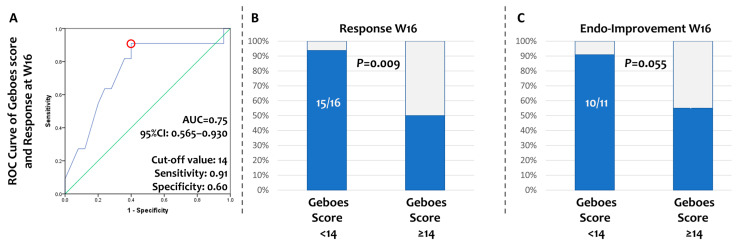
Histological severity at baseline predicts clinical and endoscopic responses to induction therapy with ustekinumab (wk 16). (**A**) Receiver operating characteristic curve of continuous Geboes score for the prediction of clinical response to ustekinumab. Continuous Geboes score of 14 was identified as the optimal cut-off value. (**B**) Patients with a continuous Geboes score of less than 14 achieved higher rates of clinical response. (**C**) The cut-off value of the Geboes score efficiently distinguishes patients with or without endoscopic improvement at W16.

Finally, we searched whether histological activity before UST initiation can predict CR at W16. The Geboes scores and sub-scores of our patients’ baseline biopsies are shown in Supplementary Table S3. Moderate-to-severe architectural changes were observed in most biopsies (69%); a moderate and marked increase in chronic inflammatory infiltrate was found in most cases (73%), while the majority (66%) had no or mild increase in lamina propria eosinophils and a mild-to-moderate increase in neutrophils (84%). Neutrophils in the epithelium with >50% crypt involvement were observed only in 18% of the obtained biopsies, while crypt destruction was either absent or local in the majority (62%) and unequivocal in 34%. Only 34% had no erosion or ulceration present at baseline biopsies. With binary logistic regression, a higher continuous Geboes score at baseline was found to be associated with a lower probability of CR at W16 (OR = 0.80, 95%CI: 0.64–0.99, *p* = 0.045). To identify a cut-off value for the baseline continuous Geboes score that could predict patients who achieved CR at the end of induction, we performed an ROC curve analysis (AUC = 0.75, 95% CI:0.57–0.93). Based on the curve and coordination table, we identified a continuous Geboes score = 14 as the value with the optimal combination of specificity (0.60) and sensitivity (0.91) for discriminating patients who achieve CR or not (Figure 4A). Patients with a continuous Geboes score of less than 14 achieved higher rates of CR (chi-square *p* = 0.009) (Figure 4B). The same cut-off (14) was also able to separate patients with or without EI at W16 (chi-square *p* = 0.055) (Figure 4C).

## 4. Discussion

We present, herein, real-world evidence of the efficacy of UST as an induction treatment for patients with active UC. We report high rates of patient- and clinician-reported outcomes 16 weeks after the commencement of the UST administration, including CR, SFCRem, and EI. We show that such outcomes can be predicted using simple baseline and on-treatment clinical and laboratory markers. We demonstrate a drug exposure/efficacy correlation, thus highlighting the importance of obtaining adequate serum UST levels. Finally, we provide insights into the mechanism of action of UST by showing that it is highly beneficial for patients with increased baseline mucosal expression of IL-23/IL-23R with no dependence on the expression of IL-12 and its receptors.

Our results support the high efficacy of UST for the treatment of patients with active UC. In our cohort, we observed a 71% CR rate and a 48% SFCRem rate at W16. In the regulatory UNIFI trial, the CR rate was 77.6% at W16 [6], whereas in real-world cohorts, post-induction response rates between 47 and 77% have been reported [13]. In the GETAID real-world cohort, CR was achieved in 53.4% in week 12–16 [14]. In Hong SJ et al.’s study, 48.9% of patients had CR at 3 months [15]. In the ENEIDA registry, it was 53% [16], and in Alsoud D. et al.’s study, 64% had CR at W16 [17]. Most likely, the differences between studies are due to variable definitions of CR. It should be mentioned that, in our study, we used a more stringent definition of CR (50% decrease in PROs from baseline) compared to other published work (oftentimes a 30% decrease). Despite this, the response rates reported herein are similar to, if not better than, other published cohorts. Furthermore, more than 70% of enrolled patients in our study had already been exposed to advanced therapies. This is in line with most real-world studies, whereby UST is used mainly as a second or third-line treatment [14,15,16,17,18]. Taken together, these results clearly validate the use of UST in the treatment of UC, including cases refractory to advanced therapy.

Recent guidance on treatment goals in UC calls for coupling clinical outcomes with objective evidence of the elimination of inflammation. The latter, at present, requires endoscopic or mucosal healing: that is, the disappearance of endoscopically active disease [19]. Our findings support the notion that treatment with UST fulfils such criteria for a treat-to-target approach, as EI was documented in most of our patients with available data on W16. Similarly to our work, Dahham Alsoud et al. reported EI in 50% of patients by W16 [17], and Honap S. et al. reported 43.8% by W26 [20]. Importantly, a Mayo score of 0 was only accepted for defining MH in our study, as it has been shown to be a much more reliable indicator of long-term benefits compared to Mayo 1. Despite this strict definition, almost one out of three patients in our cohort accomplished early MH after induction therapy with UST. This percentage is similar to the one reported by Dahham Alsoud et al. [17], thus demonstrating that hard endpoints are achievable with UST, even in a difficult-to-treat population. More importantly, we report herein that endoscopic outcomes are dependent on the drug trough levels at W16. First, patients with endoscopic response had higher UST serum concentrations as compared to non-responders. Second, there was a dose-dependent association between UST levels and the percentage of patients with endoscopic response. Finally, we were able to identify a threshold concentration of 3500 ng/mL that reliably separated endoscopic responders from non-responders in our cohort. Notably, the same cut-off value for MH was reported in the study by Jessica Petrov et al. [21]. Using other endpoints, Dahham Alsoud et al. reported a cut-off for CR of 5900 ng/mL at W8 [17]. The higher value in this study may be explained by the fact that measurements were much closer to the IV induction dose of UST. Also, in Dahham Alsoud et al., a UST threshold of 8400 ng/mL at W8 was reported to be associated with histo-endoscopic improvement by W16 [17]. This makes biological sense, as higher concentrations are expected to be required for the achievement of more demanding endpoints, such as histo-endoscopic or deep remission. Although these cut-offs have been derived from small-sample studies and should definitely be confirmed in larger cohorts with a prospective design, our findings and those from the aforementioned studies clearly emphasize the existence of an exposure/effect phenomenon during treatment with UST and may be of clinical use in the future. For example, the discovery of a cut-off value for UST concentration that predicts EI can be used to recognize patients who will benefit from dose escalation as early as W16.

One important aspect of our study is the discovery of clinical and laboratory parameters at baseline that may predict CR after induction therapy with UST in patients with UC. Firstly, a higher platelet count at baseline was a positive predictor of CR at W16. An elevated platelet count is an indicator of active inflammation and, thus, may define the subset of patients who are amenable to anti-inflammatory therapies. Similar dependence of the therapeutic effect on baseline inflammation has been previously shown. More interestingly, a decrease in platelet count at W8 was found to favor CR at W16, which can be interpreted as a measure of the effective anti-inflammatory action of UST. Therefore, serial monitoring of platelet counts may offer a simple biomarker for estimating early UST efficacy in clinical practice. Secondly, concomitant treatment with 5-ASA had a positive effect on achieving CR by W16, which is a novel finding of our study. One explanation for this may be that 5-ASA offers an added benefit in improving PROs like rectal bleeding, which are the denominators of clinical outcomes at W16. From another perspective, 5-ASA may compensate for the slow onset of UST, which may take place in certain cases. This cannot be generalized, however, as a rapid onset of action as early as 7 days after the initial dose of UST may also be observed [22]. Current clinical practice advises against the continuation of 5-ASAs in patients commencing advanced therapy. In contrast, our findings support the extension of 5-ASA administration at least during the induction phase of UST treatment in patients with UC. Finally, patients with a higher PRO/stool frequency score were less likely to respond at W16. This may indicate that increased disease burden may minimize the possibility of response and aligns with a recent study by Andres J Yarur et al. in which a higher baseline pMayo score was negatively associated with achieving SFCRem and biochemical remission [18]. Further support for this is derived from our histological study and included in our present work. In fact, we found that a continuous Geboes score of 14 or higher is related to poor CR and endoscopic outcomes. This finding is supported in a study by A. Saifuddin et al., in which the increased total Geboes score, as well as increased epithelial neutrophil sub-score, can predict resistance to UST treatment, lowering the probability of CR [23]. Taken together, our findings demonstrate a negative association between higher inflammatory activity (both clinical and histological) at baseline and lower probability of CR to induction treatment with UST.

UST is a monoclonal antibody that binds to the p40 chain that is shared by IL-23 and IL-12, thus preventing signaling via either IL-12R or IL-23R. The exact mechanism through which the anti-inflammatory effect of UST is mediated remains largely unknown at present. Our findings, however, support the notion that IL-23/IL-23R pathways are critical for the biological action of UST, since response was primarily seen in mucosal IL-23 and IL-23R mRNA over-expressors. Our work confirms previous reports of an association between the reduced expression of IL-23 and IL-23R in tissue samples at baseline endoscopy and non-response to UST [24,25]. On the other hand, expression of IL-12 and IL-12R mRNA did not affect the outcome of UST administration in our study. Those data may have important implications. From the pathophysiological standpoint, they indicate the exact stages of naïve CD4 cell differentiation that should be targeted for the treatment of ulcerative colitis. UST aimed to target both the initial stages of TH1 (through IL12) and the later stages of TH17 differentiation (through IL23). The benefit of targeting the later stages of TH1 response for the treatment of UC through the blocking of TNFa is undeniable, but its earlier targeting with UST does not seem to exert similar effects. On the contrary, selective targeting of IL23 has shown superior effectiveness for the treatment of Crohn’s disease [26]. From a translational perspective, they may establish a useful mucosal biomarker that may help select patients with an increased possibility of response to UST. Finally, from a clinical angle, our findings regarding the effect of baseline IL12 and IL23, and their receptors’ baseline expression, offer a potential mechanistic explanation for the superiority of selective IL23 targeting found in newer biological agents that specifically target the p19 chain, thus selectively inhibiting the IL-23/IL-23R pathway without affecting IL-12/IL-12R signaling [27].

Our study has certain strengths that overcome its limitations. Most importantly, its prospective design diminishes the potential collection bias, while its real-world setting differentiates our results from those of the clinical trials, making them more important in decision-making. Its multi-centric character, aiming to include the whole Greek region, and the inclusion of a relatively equal proportion of biologic-naïve patients or those exposed to 1 and ≥2 biologic agents both add to the generalizability of our results. On the other hand, half of our patients were reluctant to undergo a follow-up endoscopy in such a short period, and expression analysis and measurement of the trough levels were performed only in a sub-population. These facts make our results less representative of the whole cohort. Unfortunately, fecal calprotectin measurement has a high cost and is not reimbursed by the Greek healthcare insurance system; thus, it was not feasible to obtain longitudinal data on the biochemical response. Furthermore, we acknowledge that our study’s findings may not correspond to a number of countries with limited resources regarding treatment with biologic agents in IBD and limited access to follow-up procedures.

## 5. Conclusions

In conclusion, UST, regardless of the history of exposure to biologics, demonstrates high rates of CR, SFCRem, and EI at the end of induction in patients with UC. Lower disease burden, both clinical and histological at baseline, a decrease in platelet count at week 8, and the concomitant use of 5-ASA during the induction phase predict a more favorable outcome. Higher serum concentrations of UST at W16 are associated with better endoscopic responses, with a cut-off value of 3500 ng/mL. Patients with mucosal overexpression of IL-23/IL-23R are more likely to achieve CR at the end of induction. Collectively, our findings support the feasibility of identifying clinical, laboratory, and molecular biomarkers with a predictive value for response to advanced therapies in IBD. Such recognition may serve as a starting point for the development of a personalized management of patients.

## Figures and Tables

**Table 1:** Characteristics of 123 included patients.

Age in Years [Mean (SD)]	50.3 (18)
Male sex [n (%)]	78 (63.4)
Montreal Classification [n (%)]	
E1	2 (1.6)
E2	56 (45.5)
E3	65 (52.8)
Disease duration in years [median (IQR)]	8 (3–15)
Family history [n (%)]	9 (7.3)
Extra-intestinal manifestations [n (%)]	39 (31.7)
Articular	29 (23.6)
Skin	11 (8.9)
Liver	2 (1.6)
Ocular	1 (0.8)
Other	5 (4.1)
Previous treatments [n (%)]	
Corticosteroids	106 (86.2)
5-ASA	118 (95.9)
Thiopurines	46 (37.4)
Methotrexate	5 (4.1)
Anti-TNF	59 (48%)
Infliximab	51 (41.5)
Adalimumab	18 (14.6)
Golimumab	8 (6.5)
Vedolizumab	53 (43.1)
Tofacitinib	13 (10.6)
Number of previous biologics [n (%)]	
0	36 (29.3)
1	49 (39.8)
2	25 (20.3)
≥3	13 (10.6)
Concomitant treatments [n (%)]	
Corticosteroids	62 (50.4)
Mesalazine	85 (69.1)
Thiopurines	12 (9.8)
Methotrexate	1 (0.8)
C-reactive protein mg/L [median (IQR)]	3.0 (0.96–10.2)
Albumin, g/dL [mean (SD)]	4.2 (3.8–4.5)
Hemoglobin, g/dL [mean (SD)]	13.3 (12.2–14.5)
Platelets, 10^9^/L [mean (SD)]	294 (239–354)
WBC, 10^6^/L [mean (SD)]	8115 (6408–10,863)
Endoscopic MAYO score [median (IQR)]	2 (2–3)
UCEIS [median (IQR)]	5 (4–6)

SD, Standard deviation; IQR, interquartile range; WBCs, white blood cells; Ulcerative Colitis Endoscopic Index of Severity (UCEIS).

**Table 2:** Baseline predictors of clinical response at week 16.

	Univariate			Multivariate		
	OR	95% CI	*p*	OR	95% CI	*p*
Steroids experienced	0.17	0.02–1.37	0.096	0.30	0.03–2.87	0.298
Vedolizumab experienced	0.45	0.19–1.07	0.069	0.61	0.22–1.65	0.327
PRO-Stool frequency	0.61	0.37–1.02	0.058	0.49	0.26–0.92	0.027
Concomitant 5asa	2.72	1.10–6.77	0.031	3.69	1.2–11.17	0.021
Concomitant steroids	0.37	0.15–0.93	0.053	0.47	0.16–1.38	0.170
Platelets ≥ 284 × 10^9^/L	3.21	1.31–7.86	0.011	6.52	2.08–20.46	0.001

**Table 3:** Early predictors of clinical response at week 16.

	Univariate			Multivariate		
	OR	95% CI	*p*	OR	95% CI	*p*
PRO-rectal bleeding drop, per 1	2.22	1.23–4.01	0.008	1.61	0.85–3.13	0.145
Concomitant 5-ASA	2.71	0.96–7.69	0.061	1.84	0.51–6.62	0.353
Platelets’ total count drop, per 108/L	1.28	1.08–1.52	0.006	1.23	1.03–1.47	0.022

## Data Availability

The data presented in this study are available upon request from the corresponding author.

## Supplementary Materials

The following supporting information can be downloaded at https://www.mdpi.com/article/10.3390/diseases14040149/s1. Figure S1: Study flow chart of the number of patients’ data analyzed; Figure S2: Pre-treatment mucosal expression of *IL-12* and *IL-12* receptor do not impact response to therapy with Ustekinumab in ulcerative colitis; Table S1: Participant centers of the study; Table S2: Primer sequences of the genes used in RT-PCR; Table S3: Geboes score grading and subscores of biopsies acquired at baseline.

## Abbreviations

The following abbreviations are used in this manuscript:

Crohn’s disease	(CD);
Extra-intestinal manifestations	(EIMs);
Inflammatory bowel disease	(IBD);
Partial MAYO	(pMAYO);
Patient-reported outcome	(PRO);
Receiver operating characteristics	(ROC);
Real-time reverse transcription polymerase	(RT-PCR);
Ulcerative colitis	(UC);
Ulcerative Colitis Endoscopic Index of Severity	(UCEIS);
Ustekinumab	(UST).
