# Supplementary material for: Predictors of Response to Induction Therapy with Ustekinumab in Patients with Ulcerative Colitis: Results from a National Study in Greece
Source: Diseases. 2026 Apr 19;14(4):149. doi: 10.3390/diseases14040149 (PMC13115418; doi:10.3390/diseases14040149)
Supplement: Supplementary file 1 [file diseases-14-00149-s001.zip › Supplementary Figure S1.pdf]

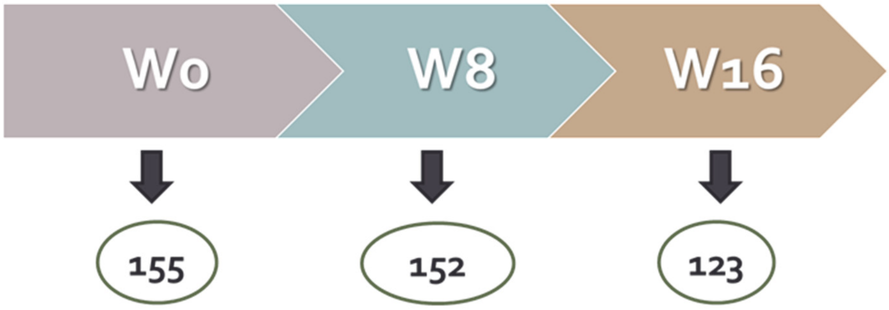

Supplementary Figure S1. ***Study flow chart of the number of patients' data analyzed***. *Three patients were excluded due to loss of follow-up at week 8. At week 16 loss of follow-up or inadequate data were recorded in 32 patients, who were excluded from further analysis.*
