# Supplementary material for: Predictors of Response to Induction Therapy with Ustekinumab in Patients with Ulcerative Colitis: Results from a National Study in Greece
Source: Diseases. 2026 Apr 19;14(4):149. doi: 10.3390/diseases14040149 (PMC13115418; doi:10.3390/diseases14040149)
Supplement: Supplementary file 1 [file diseases-14-00149-s001.zip › Supplementary Figure S2.pdf]

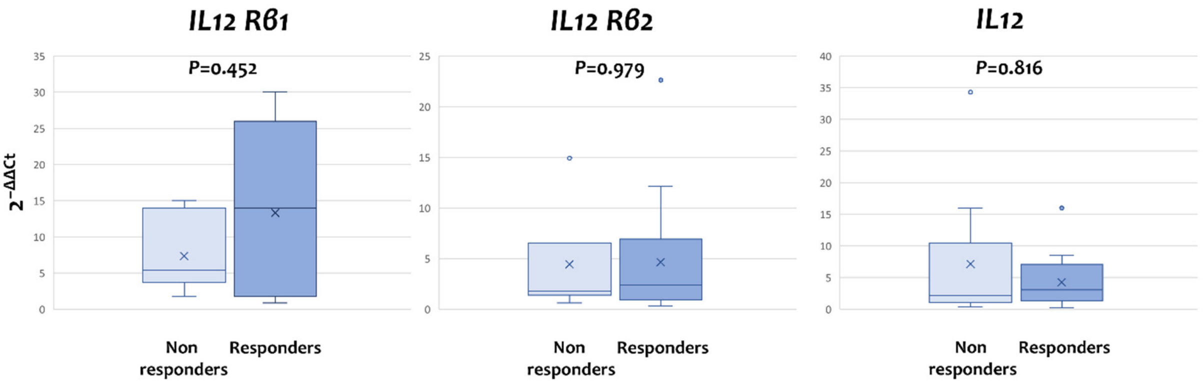

Supplementary Figure S2. ***Pre-treatment mucosal expression of IL-12 and IL-12 receptor do not impact response to therapy with Ustekinumab in ulcerative colitis.*** *The mRNA expression for IL-12 IL-12Rβ1 and IL-12Rβ2 was quantified in total RNA extracted from endoscopically obtained biopsies before treatment with ustekinumab.*
