# Supplementary material for: Predictors of Response to Induction Therapy with Ustekinumab in Patients with Ulcerative Colitis: Results from a National Study in Greece
Source: Diseases. 2026 Apr 19;14(4):149. doi: 10.3390/diseases14040149 (PMC13115418; doi:10.3390/diseases14040149)
Supplement: Supplementary file 1 [file diseases-14-00149-s001.zip › Supplementary Table S1.pdf]

Supplementary Table S1. *Participant centers of the study*

	Participant centers of the study
**1.**	3rd Academic Department of Internal Medicine, University of Athens- Sotiria Hospital- GI Unit, Athens, Greece
**2.**	Attikon University Hospital, Department of Gastroenterology, Athens, Greece
**3.**	General Hospital G. Gennimatas, Department of Gastroenterology, Athens, Greece
**4.**	Aristotle University of Thessaloniki, 2nd Propaedeutic Department of Internal Medicine, Thessaloniki, Greece,
**5.**	Evangelismos Hospital, Department of Gastroenterology, Athens, Greece
**6.**	Theagenio Cancer Hospital of Thessaloniki, Department of Gastroenterology, Thessaloniki, Greece,
**7.**	General Hospital Laiko, Department of Gastroenterology, Athens, Greece
**8.**	General Hospital of Nikea and Pireaus, Department of Gastroenterology, Athens, Greece
**9.**	General Hospital Alexandra, Department of Gastroenterology, Athens, Greece
**10.**	Venizeleio General Hospital, Department of Gastroenterology, Heraklion- Crete, Greece
**11.**	General University Hospital of Patras, Department of Gastroenterology, Patras, Greece
**12.**	Saint George General Hospital of Chania, Department of Gastroenterology, Chania- Crete, Greece
**13.**	University General Hospital of Larissa, Department of Gastroenterology, Larissa, Greece
**14.**	University General Hospital of Heraklion, Department of Gastroenterology, Heraklion- Crete, Greece
**15.**	401 General Military Hospital, Department of Gastroenterology, Athens, Greece
**16.**	Metaxa Cancer Hospital of Athens, Department of Gastroenterology, Athens, Greece
