# Supplementary material for: Predictors of Response to Induction Therapy with Ustekinumab in Patients with Ulcerative Colitis: Results from a National Study in Greece
Source: Diseases. 2026 Apr 19;14(4):149. doi: 10.3390/diseases14040149 (PMC13115418; doi:10.3390/diseases14040149)
Supplement: Supplementary file 1 [file diseases-14-00149-s001.zip › Supplementary Table S2.pdf]

Supplementary Table S2. *Primer sequences of the genes used in RT-PCR*

Gene	Primer sequences
IL12RB1	5′-CAGTGGCTCTGAATATCAGC-3′
	5′-TGCACCGTGTAGGCTACACC-3′
IL12RB2	5′-GGAGTGAATCATTGAGAGCACAA-3′
	5′-TGCCGTTTCATGTACCAGAC-3′
**IL-12**	5′-AGTGTCAAAAGCAGCAGAGG-3′
	5′- AACGCAGAATGTCAGGGAG-3′
**IL-23R**	5′-ACAGTTCCCCAGGTCACATC-3′
	5′-CCCAGTTCGGAATGATCTGT-3′
**IL23**	5′-AAGTGGAAGTGGGCAGAGAT-3′
	5′-ATCCTTGAGCTGCTGCCTTT-3′
GAPDH	5′-CCCCATGTTCGTCATGGGT-3′
	5′-GTGATGGCATGGACTGTGG-3′
