# Supplementary material for: Predictors of Response to Induction Therapy with Ustekinumab in Patients with Ulcerative Colitis: Results from a National Study in Greece
Source: Diseases. 2026 Apr 19;14(4):149. doi: 10.3390/diseases14040149 (PMC13115418; doi:10.3390/diseases14040149)
Supplement: Supplementary file 1 [file diseases-14-00149-s001.zip › Supplementary Table S3.pdf]

Supplementary Table S3. Geboes score grading and subscores of biopsies acquired at baseline.

Geboes score (n=103)	
Grade 0 [n (%)]	
0	4 (4)
1	27 (26)
2	38 (37)
3	33 (32)
Grade 1 [n (%)]	
0	6 (6)
1	21 (21)
2	41 (40)
3	34 (33)
Grade 2A [n (%)]	
0	32 (31)
1	36 (35)
2	23 (23)
3	11 (11)
Grade 2B [n (%)]	
0	11 11)
1	50 (49)
2	36 (35)
3	6 (6)
Grade 3 [n (%)]	
0	16 (15)
1	29 (28)
2	39 (38)
3	19 (18)
Grade 4 [n =99, (%)]	
0	49 (49)
1	13 (13)
2	3 (3)
3	34 (34)
Grade 5 [n (%)]	
0	32 (31)
1	6 (6)
2	19 (18)
3	13 (13)
4	33 (32)
